# Draft genome sequence of *Lentzea* sp. H45 from high-altitude Atacama Desert soil

**DOI:** 10.1128/mra.01122-25

**Published:** 2026-04-15

**Authors:** Weijie Zhou, Jenileima Kshetrimayum Devi, Nick Allenby

**Affiliations:** 1 John Dawson Drug Discovery Centre, University of Sunderland 7735, Sunderland, United Kingdom; University of Wisconsin-Madison, Madison, Wisconsin, USA

**Keywords:** *Lentzea*, extremophile, Atacama Desert, secondary metabolites, genome sequence, lentzeosides

## Abstract

We report the draft genome sequence of *Lentzea* sp. H45, an extremotolerant actinobacterium from high-altitude Atacama Desert soil, producing anti-HIV-1 integrase lentzeosides. The genome comprises 8.7 Mbp with 70.9% GC content and harbors 34 biosynthetic gene clusters, including a putative oligosaccharide cluster for lentzeoside biosynthesis.

## ANNOUNCEMENT

*Lentzea* sp. H45 was isolated from a high-altitude soil sample (5,000 m above sea level) collected near Cerro Chajnantor in the Atacama Desert, northern Chile, using selective actinomycete isolation media as previously described (1). The strain was previously characterized as the producer of six novel anti-HIV-1 integrase compounds, lentzeosides A–F (1). This strain represents a phylogenetically distinct lineage within the genus *Lentzea* and highlights the potential of extremotolerant actinomycetes from the Atacama Desert as a source of novel specialized metabolites (1, 2). However, the whole genome sequence of this strain has not been reported. Here, we report the draft genome sequence of strain H45, providing a genomic resource that complements its chemical characterization and enables future exploration of its biosynthetic potential.

**Fig 1:**
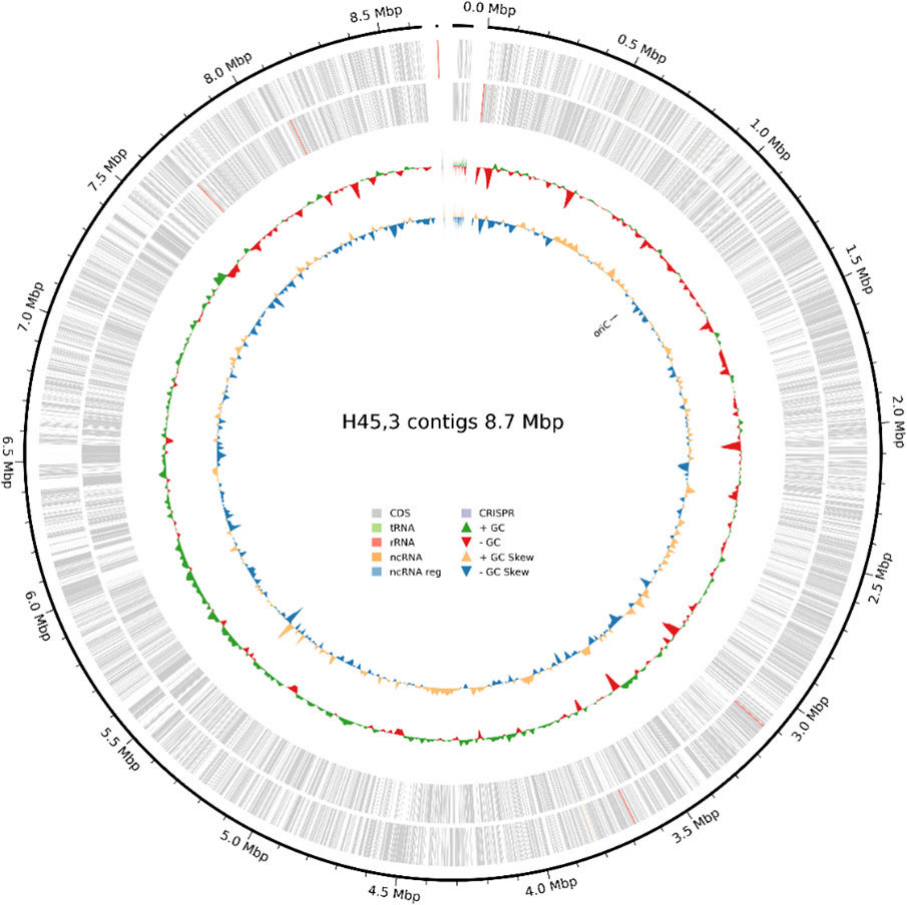
Circular genome map of *Lentzea* sp. H45.

*Lentzea* sp. H45 was maintained as glycerol stocks at −80°C and cultivated on ISP2 agar at 30°C for 48 h (3). Mycelial biomass was collected by gently scraping the surface of the agar plates with a sterile loop and suspended in DNA/RNA Shield solution (Zymo Research, USA) for preservation. The harvested cells were stored at −20°C until further processing. High-molecular-weight genomic DNA was isolated using the Quick-DNA HMW MagBead kit (Zymo Research, USA), following the manufacturer’s protocol. DNA concentration and purity were measured using a Qubit 3.0 fluorometer (Invitrogen, USA), and the integrity of the extracted DNA was verified by agarose gel electrophoresis (4). Library preparation was carried out using the Ligation sequencing V14 kit with native barcoding expansion (SQK-NBD114.24; Oxford Nanopore Technologies [ONT], UK), following the manufacturer’s protocol (5). Four hundred nanograms of DNA was used in the end-repair reaction, sequencing adapters were ligated, and the final library was sequenced using a MinION Flow Cell (ONT, UK). Basecalling was performed in real time using the MinKNOW platform (Oxford Nanopore Technologies, UK). Sequencing generated a total of 169,574 reads with a read N50 of 4,306 bp, a mean read quality of Q17.7, and a total yield of 541.2 Mb. Genome assembly was performed using Flye v2.9.5 (**--nano-raw**) (6). The resulting assembly was polished with Medaka v1.11.3 (https://github.com/nanoporetech/medaka) (ONT, **-t 12, -b 100, default ONT model**). The polished assembly was then annotated with Bakta v1.10.1 (--db full Bakta database v5.1, --force) (7). Genome completeness and contamination were assessed using CheckM v1.2.2 with the lineage-specific workflow (8), showing 97.3% completeness with 1.9% contamination, indicating a high-quality draft genome. The draft genome assembly consists of three contigs with an N50 value of 8,639,429 bp and a total length of 8,712,140 bp (Table 1). The genome has a G + C content of 70.9%, which is consistent with other members of the genus *Lentzea*. A circular genome map of *Lentzea* sp. H45 is shown in Fig. 1.

**TABLE 1:** General genome features of *Lentzea* sp. H45

Category	Feature	Value
Strain	Strain	H45
	Species	*Lentzea* sp.
Sequencing statistics	Total reads	169,574
	Sequencing yield (Mb)	541.2
	Mean read length (bp)	3,192
	Median read length (bp)	2,392
	Read N50 (bp)	4,306
	Mean read quality (*Q*)	17.7
	Reads >Q10 (%)	99.8
Assembly statistics	Genome size (bp)	8,712,140
	Contigs	3
	Assembly N50 (bp)	8,639,429
	GC content (%)	70.9
	Coverage (×)	57
	Completeness (%)	97.3
	Contamination (%)	1.9
Annotation statistics	CDS	8,152
	tRNA	61
	rRNA	17

Analysis of secondary metabolite biosynthetic gene clusters (BGCs) was performed using antiSMASH v7.1.0 (**--cc-mibig, --cb-general, --cb-knownclusters, --cb-subclusters)** (9). A total of 34 BGCs were identified, including 7 NRPS/NRPS-like clusters, 8 PKS clusters (4 Type I, 2 Type II, and 1 Type III), 4 terpene clusters, 3 lanthipeptide clusters, 2 indole clusters, 1 oligosaccharide cluster, and 10 other types of specialized clusters. Several BGCs showed high similarity to known bioactive compounds, including clusters for ε-poly-L-lysine (100% similarity), chloramphenicol-related compounds (88% similarity), geosmin (100% similarity), and tripartilactam/niizalactam C (92% similarity). Most importantly, we identified a 40.7 kb oligosaccharide BGC that likely represents the lentzeoside biosynthetic machinery responsible for the production of the previously characterized anti-HIV-1 glycosides (1). Although the cluster shows only low similarity (3%) to known polyketides such as LL-D49194α1, this is expected as lentzeosides represent a novel class of diene/monoene glycosides with unique structural features. The identification of 34 BGCs, including the putative lentzeoside biosynthetic pathway, demonstrates the significant natural product potential of the extremotolerant strain.

## Data Availability

The draft genome sequence of *Lentzea* sp. H45 has been deposited in NCBI under BioProject PRJNA1333581, BioSample SAMN52068101, and GenBank accession JBSDZT000000000. Raw sequencing reads are available in the Sequence Read Archive (SRA) under accession SRR35636518.

## References

[1] Wichner D, Idris H, Houssen WE, McEwan AR, Bull AT, Asenjo JA, Goodfellow M, Jaspars M, Ebel R, Rateb ME. 2017. Isolation and anti-HIV-1 integrase activity of lentzeosides A–F from extremotolerant lentzea sp. H45, a strain isolated from a high-altitude Atacama Desert soil. J Antibiot 70:448–453. doi:10.1038/ja.2016.78 27353167

[2] Idris H, Nouioui I, Asenjo JA, Bull AT, Goodfellow M. 2017. Lentzea chajnantorensis sp. nov., an actinobacterium from a very high altitude Cerro Chajnantor gravel soil in northern Chile. Antonie Van Leeuwenhoek 110:795–802. doi:10.1007/s10482-017-0851-5 28324230

[3] Shirling EB, Gottlieb D. 1966. Methods for characterization of streptomyces species. Int J Syst Bacteriol 16:313–340. doi:10.1099/00207713-16-3-313

[4] Sambrook J, Russell DW. 2001. Molecular Cloning: Ch. 8. In vitro amplification of DNA by the polymerase chain reaction. Cold Spring Harbor Laboratory Press.

[5] Macpherson H, Gustavsson E, Lee J. 2023. Native barcoding (SQK-NBD114) gDNA for adaptive sampling using oxford nanopore technologies

[6] Kolmogorov M, Bickhart DM, Behsaz B, Gurevich A, Rayko M, Shin SB, Kuhn K, Yuan J, Polevikov E, Smith TPL, Pevzner PA. 2020. metaFlye: scalable long-read metagenome assembly using repeat graphs. Nat Methods 17:1103–1110. doi:10.1038/s41592-020-00971-x 33020656 PMC10699202

[7] Schwengers O, Jelonek L, Dieckmann MA, Beyvers S, Blom J, Goesmann A. 2021. Bakta: rapid and standardized annotation of bacterial genomes via alignment-free sequence identification. Microb Genom 7:000685. doi:10.1099/mgen.0.000685 34739369 PMC8743544

[8] Parks DH, Imelfort M, Skennerton CT, Hugenholtz P, Tyson GW. 2015. CheckM: assessing the quality of microbial genomes recovered from isolates, single cells, and metagenomes. Genome Res 25:1043–1055. doi:10.1101/gr.186072.114 25977477 PMC4484387

[9] Blin K, Shaw S, Augustijn HE, Reitz ZL, Biermann F, Alanjary M, Fetter A, Terlouw BR, Metcalf WW, Helfrich EJN, van Wezel GP, Medema MH, Weber T. 2023. antiSMASH 7.0: new and improved predictions for detection, regulation, chemical structures and visualisation. Nucleic Acids Res 51:W46–W50. doi:10.1093/nar/gkad344 37140036 PMC10320115

